# Prevalence and Determinants of Iodized Salt Purchase: Findings from an In-Person Supermarket Survey in Northern Portugal

**DOI:** 10.3390/nu18152537

**Published:** 2026-08-04

**Authors:** Sarai Isabel Machado, Susana Roque, Leonor Belo Pereira, Ester Marques, João Colaço Belo, Nuno Borges, Patrício Costa, Joana Almeida Palha

**Affiliations:** 1 Life and Health Sciences Research Institute (ICVS), School of Medicine, University of Minho, 4710-057 Braga, Portugal; 2 ICVS/3B’s-PT Government Associate Laboratory, 4710-057 Braga, Portugal; 3 USF Entre Homem e Cávado, ULS Braga, 4720-393 Ferreiros, Portugal; 4 Faculty of Food Sciences and Nutrition (FCNAUP), University of Porto, 4150-180 Porto, Portugal

**Keywords:** iodized salt, iodine deficiency, consumer awareness, supermarket purchase, Portugal

## Abstract

**Background/Objectives**: Iodine is an essential micronutrient required for the synthesis of thyroid hormones, fundamental in metabolic processes throughout life. Salt iodization is recognized as the most cost-effective and recommended strategy for the prevention of iodine deficiency disorders. In Portugal, the use of iodized salt remains voluntary for the general population, dependent on consumers’ choice, where iodized salt accounts for approximately 11% of retail salt sales. This cross-sectional study aimed to evaluate prevalence, knowledge, and motives of iodized salt purchase among consumers in all stores of a major supermarket chain in Braga, Portugal. **Methods**: A questionnaire assessing purchasing behavior, knowledge, motivations, and barriers related to choosing iodized salt was administered to individuals in supermarkets after they had selected a package of salt from the shelf, during January and February of 2023. **Results**: While 21% of the 396 in-store respondents reported consuming iodized salt at home, only 10% were observed purchasing it in the store. Approximately half were unaware of the availability of iodized salt, and only 20% associated health benefits with it. Perceived health benefits were the most frequently reported motives for purchase, whereas lack of knowledge emerged as the main barrier. **Conclusions**: These findings indicate a low prevalence of iodized salt use and limited awareness of its availability and its role in preventing iodine deficiency disorders among this large urban population, consistent with reports of low iodized salt sales nationwide. Stronger public health measures, including literacy campaigns, improved product labeling and visibility, and broader policy actions such as universal salt iodization should be considered.

## 1. Introduction

Iodine is an essential micronutrient primarily found in seafood, algae, and dairy products. It is required for the synthesis of thyroid hormones, which are necessary throughout life. Given the extensive evidence of inadequate iodine intake in many populations, the World Health Organization (WHO) recommends universal salt iodization as the most cost-effective strategy to prevent iodine deficiency disorders [1]. Nearly 89% of the global population is covered by this measure. In the European region, 11 countries lack specific iodization programs or have an unknown status (e.g., the United Kingdom), and 13 have voluntary policies (e.g., Germany and Greece), leaving vulnerable groups such as pregnant women and children at risk, as reflected in the low median urinary iodine levels observed in these populations [2].

In Portugal, the policy for iodized salt is voluntary for the general population, but mandatory in school canteens since 2013 and in hospitals since 2021 [3,4]. However, compliance has been limited; a 2017 study in Northern Portugal reported no adherence among surveyed canteens [5]. More recently, legislation in the Autonomous Region of Madeira mandated the use of iodized salt in the food industry (e.g., bread manufacturing), although implementation is still pending [6]. In the Autonomous Region of Azores, iodized salt is mandatory in meal services provided by regional public administration institutions, including healthcare facilities and schools [7].

Portugal is classified as iodine-sufficient based on the median urinary iodine of school-aged children [8]. However, children may not accurately represent the general population, as their dietary patterns, particularly higher dairy consumption, may lead to adequate iodine intake. In contrast, women of childbearing age and pregnant women remain iodine deficient, despite national recommendations issued in 2013 advocating for iodine supplementation during preconception, pregnancy and lactation [9,10,11,12,13]. This deficiency is reflected in neonatal screening data, with more than 3% of newborns presenting thyroid-stimulating hormone (TSH) levels above 5 mUI/L, a recognized population indicator of iodine deficiency [14].

In response, the National Program for the Promotion of Healthy Eating for 2022–2030 has prioritized universal salt iodization and increased awareness among the public and healthcare professionals [15].

Iodized salt is rarely used in processed foods, and its usage depends on consumer choice at the household level. Despite a recent increase in sales, iodized salt accounted for only 11% of total sales in Portugal in 2022, which is insufficient to ensure adequate iodine status at the population level [16]. This highlights the importance of understanding consumers’ behavior and knowledge. Previous studies in Portugal and other European countries have consistently shown limited awareness of iodine, its dietary sources, and the benefits of iodized salt, underscoring the need for targeted health literacy interventions [17,18,19,20,21].

Given these considerations, the present study aimed to assess the prevalence of iodized salt purchase, evaluate knowledge of iodized salt, and identify factors associated with its purchase among salt buyers in Braga, Portugal, using a brief questionnaire administered in person at supermarkets.

## 2. Materials and Methods

### 2.1. Study Design and Setting

This is a cross-sectional study that was conducted between January and February of 2023 among customers in all six Braga stores (both in rural and urban areas) of a major retailer, Pingo Doce. While the retailer has a large national market share representing about 25% of the national market share of salt, the sampling frame was restricted to Braga, a major city with ~200,000 residents in Northern Portugal, the 6th largest in the country.

### 2.2. Study Participants and Sample Size Calculations

All customers aged 18 years or older who selected a salt product in the stores and who were able to understand Portuguese or English were eligible for inclusion and were invited to participate in the study. Individuals who met these criteria and provided verbal informed consent were included.

**Figure 1:**
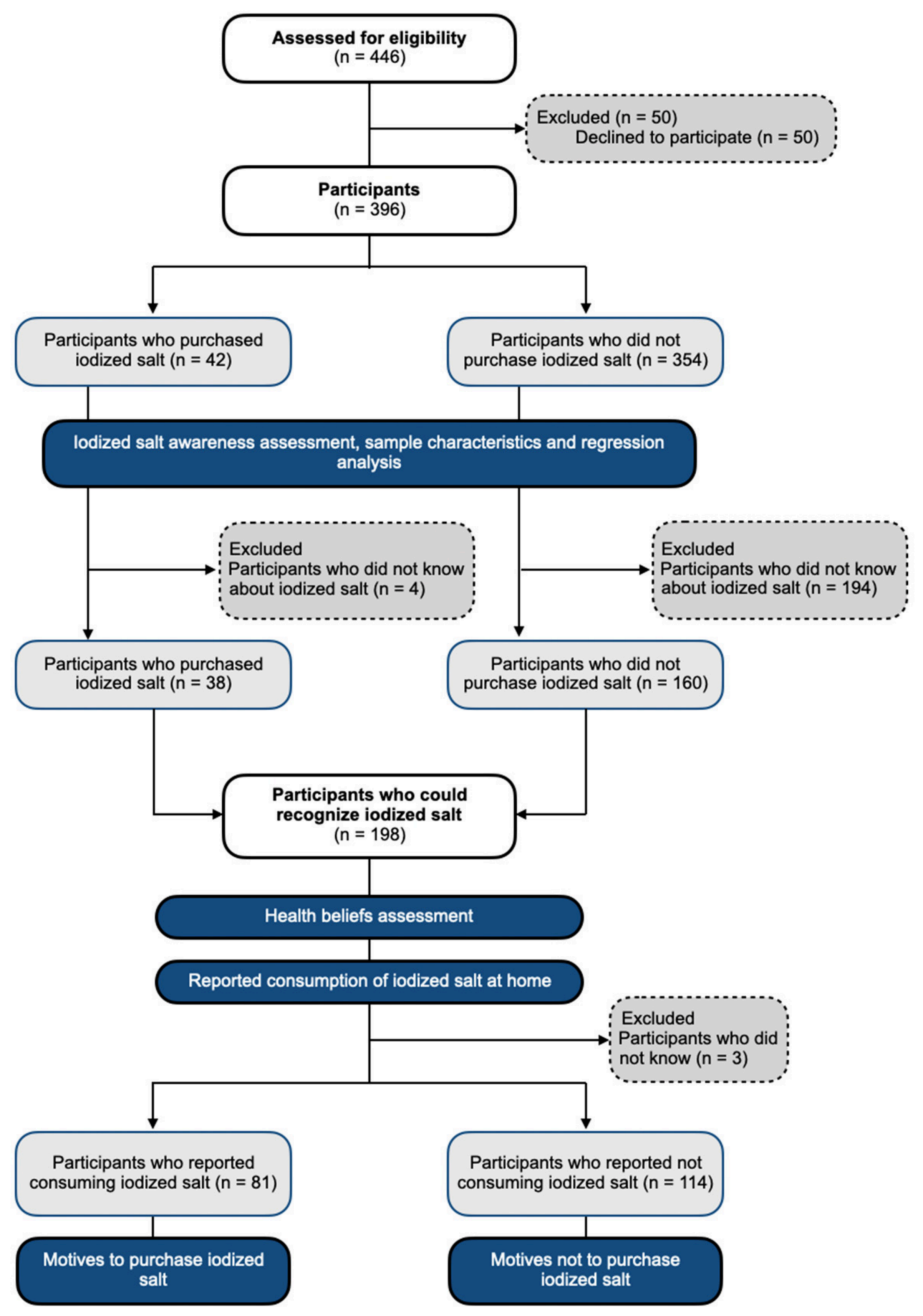
Flow diagram of study participants.

The required sample size was calculated using the OpenEpi online calculator [22]. Because no prior data were available on directly verified iodized salt purchases, we used the reported use of iodized salt as a proxy for the expected outcome frequency. The expected prevalence was set at 8%, based on a previous study in Porto (Portugal) that reported this level of household iodized salt use [5]. Using a 95% confidence level and a precision of ±3%, the minimum required sample size was therefore estimated at 315 participants. The data collection period was defined based on the estimated monthly volume of salt sales; data collection would be extended until the required number of participants was reached. In practice, this was not necessary, as from the 446 customers approached, 396 agreed to participate (participation rate of 89%). A flow diagram of the study participants, including sample size for each analysis, is presented in Figure 1.

### 2.3. Assessment of the Main Outcome

Prevalence of iodized salt purchase was the study outcome of interest and was estimated by dividing the number of observed purchasers of this type of salt among all salt purchasers. Actual purchase was verified through direct observation (physical verification of the salt package label) by the respective investigator who directly asked participants to show the type of salt they selected from the shelf. Data collection was conducted by three investigators on randomly selected days and time periods throughout the study period. For each store, observations were scheduled to include two weekdays and one weekend day, with coverage of all operating hours (08:00–21:00 h).

### 2.4. Assessment of the Main Determinants

Assessment of potential determinants of iodized salt purchase was conducted with the use of an interviewer-administered questionnaire, the design and structure of which was informed by similar studies [17,21]. Specifically, the questionnaire included items for sociodemographic characterization of the respondents (i.e., sex, year of birth, education level, and presence of any ongoing pregnancy or recent birth among the circle of friends and/or family), and questions addressing unaided recall of types of salt (top-of-mind), knowledge (recognition) of types of salt, and the type of salt chosen for purchase. Responders who knew about iodized salt were further asked about habitual consumption at home, motives for purchasing it or not, and beliefs about the health benefits of iodized salt.

Health beliefs regarding iodized salt were assessed by measuring participants’ level of agreement with six statements using a 5-point Likert scale (1—“strongly disagree”, 2—“disagree”, 3—“neither agree nor disagree”, 4—“agree”, 5—“strongly agree”). Of these, three statements reflected established evidence-based health benefits of iodized salt and the most common health concerns and frequently asked questions in previous questionnaires (i.e., “Iodized salt contributes to the psychomotor development of children”, “Iodized salt contributes to the normal functioning of the thyroid” and “Iodized salt contributes to preventing goiter”). The other three statements represented false associations to enable differentiation of respondents: two random health conditions in which iodized salt has no role (“Iodized salt contributes to preventing asthma” and “Iodized salt contributes to protection from sunburn” and one situation where specific iodine supplements may be indicated but iodized salt has no role (“Iodized salt contributes to protection in nuclear accidents”).

The internal consistency of the health beliefs assessment was evaluated using Cronbach’s alpha. Scores for false statements were reverse-coded before the analysis, yielding a value of 0.7, which indicates an acceptable level of internal consistency reliability.

Content validity, including relevance, comprehensiveness, and comprehensibility, was assessed in a pilot study targeting both experts and members of the general population (n = 33). Feedback obtained during this phase was used to improve the wording, clarity, and overall comprehensibility of the questionnaire.

The questionnaire was applied in Portuguese or English, if necessary. The English-translated version is presented in Appendix A (Table A1).

### 2.5. Data Analysis

For descriptive statistics, the normality of the distribution of continuous variables was evaluated using the Kolmogorov–Smirnov test, as well as measures of skewness and kurtosis and visual inspection of histograms. Means (M) and standard deviations (SD) were calculated for variables that met the normality assumption, while medians (Md) and percentiles 25 (P25) and 75 (P75) were calculated for variables that did not meet this assumption. Categorical variables were described with absolute and relative frequencies. Comparisons of variable means, medians or frequencies between two groups (purchasers vs. non-purchasers of iodized salt) were conducted with Student’s t, Mann–Whitney and Chi-square tests, respectively.

Simple and multiple logistic regression models were used to examine whether age, sex, education, purchasing salt as a habitual choice (given that habit was reported as a major reason for selecting a particular type of salt), responsibility for household salt purchases (as this may reflect the person delegated to make purchasing decisions), having a pregnant or postpartum woman in one’s close social circle (as health professionals may provide information on iodine nutrition during pregnancy), and perceived health benefits of iodized salt were associated with the actual purchase of iodized salt.

Qualitative assessment of all responses was independently performed by two researchers. Discrepancies in categorization were reviewed and resolved through discussion with a third researcher.

Goodness of fit was evaluated using the Hosmer-Lemeshow test. Statistical analysis was performed using IBM SPSS Statistics version 29.0.0.0. Statistical significance was set at *p* < 0.05. The manuscript was prepared using Microsoft Word for Mac (version 16.103.1) and a study flowchart was constructed using Keynote (version 14.4).

### 2.6. Ethics

The study was approved by the Ethical Committee for the Health Sciences of the University of Minho (CEICVS 134/2022).

## 3. Results

A total of 446 customers were observed selecting a salt package from the supermarket shelves and were approached; 396 agreed to participate (participation rate of 89%). General characteristics of the participants are described in Table 1. Customers’ mean age was 51 years (SD = 15), 82% were women, and 59% had secondary education or higher. Only 10% selected an iodized salt product during the observed supermarket purchase (actual purchase), though 21% of customers reported usually consuming iodized salt at home. Twenty percent attributed health benefits to iodized salt. Regular coarse salt was the most common choice of salt (45%).

Analysis of the predictors of actual iodized salt purchase indicated that among the variables examined, only age and the attribution of health benefits to iodized salt were independent predictors of its purchase (Table 2).

Those who attributed health benefits to iodized salt presented 8.8-fold higher odds of purchasing iodized salt compared to customers who did not know about the benefits or did not attribute any benefit. Each additional year of age was associated with 4% higher odds of iodized salt purchase. Sex, education, pregnancy/postpartum in close circle, responsibility for salt purchase, and usual choice did not influence purchasing behavior. The Hosmer-Lemeshow test indicated that the model was a good fit to the data, *χ*^2^(8) = 4.89, *p* = 0.769. A sensitivity analysis using a reduced model that included only the predictors considered most influential: age, sex, education, responsibility for household salt purchases, and perceived health benefits of iodized salt, provided identical results.

Table 3 describes consumers who recognized iodized salt regarding their health benefits beliefs. Of note, four respondents who purchased iodized salt were unable to identify it as such, and therefore the additional questions were not applied to them, resulting in a final sample of 38 assessed purchasers for the following assessments.

Regarding the associated health beliefs, only the sentence “Iodized salt contributes to normal thyroid function” presented a median of 4 (corresponding to the answer “agree”) within iodized salt buyers, while medians in all others were 3 (corresponding to “do not agree, nor disagree”).

To the 81 consumers who reported consuming iodized salt at home, the most common motive for purchase was the associated health benefits (62%) (Table 4).

To the 114 consumers who knew about iodized salt, but reported not consuming iodized salt at home, the most common deterrents for not purchasing iodized salt were lack of knowledge of its characteristics (25%) and the habit of purchasing other types of salt (22%) (Table 5).

## 4. Discussion

The findings of this study indicate a low prevalence of iodized salt purchase (10%) among the population of Braga (mainland Portugal), accompanied by limited knowledge of its existence (50%) and of the health benefits of iodized salt (20%). Interestingly, 21% of participants reported consumption of iodized salt at home. This difference between the reported use of iodized salt and actual acquisition was also described in a study in the city of Porto, Portugal, where 8% reported being purchasers and only 2% actually had iodized salt at home [5]. The discrepancy may be attributed to social desirability bias and, in some cases, to the belief that all salt is iodized. This belief was particularly reported by respondents whose countries of origin have mandatory salt iodization practices, such as Brazil. These findings indicate the need for improved labeling of salt products to clearly highlight their composition. Overall, the actual purchase rate (10%) aligns with iodized salt sales data in Portugal [16], and remains far below the 89% of households using iodized salt worldwide and the 90% coverage target by the WHO [1].

Knowledge of iodized salt existence (50%) is lower than that previously reported in the Autonomous Region of Azores (68%) [17], but higher than that identified by a nationwide survey on iodine supplementation in school-age children (17%) [23].

Knowledge of the health benefits of iodine was present in only 60% of the iodized salt purchasers compared with 15% of the non-iodized salt purchasers. The relation between awareness of iodized salt and actual purchase may be compromised by this poor understanding of iodine’s health benefits, as reported in studies in European and Asian countries [21,24]. A study conducted in 2022 in Germany and Greece, countries with voluntary salt iodization policies (similar to Portugal), reported iodized salt use among students of 41% and 37%, respectively, and found that greater knowledge of iodized salt was associated with increased use [21,24]. These findings prompted action, with Germany launching a campaign to raise awareness of the importance of iodine for health in 2023 [25].

Studies assessing knowledge among women of childbearing age have reported limited awareness of dietary iodine sources and indicate that antenatal care often provides insufficient information on this topic [26]. Similarly, findings from 16 countries across America, Europe, Asia, and Oceania showed substantial gaps in knowledge of dietary iodine sources, despite general awareness of iodine’s role in health [27]. Furthermore, a Delphi study identified insufficient knowledge and information as key barriers to the elimination of iodine deficiency disorders, emphasizing the need for targeted interventions to improve population awareness as a public health priority [28].

In the present study, when inquired about the health benefits of iodine using statements that included both true and false claims, knowledge was very low, with the neutral response (“neither agree nor disagree”) being predominant. The exception was the correct association of iodine with normal thyroid function among iodized salt purchasers. Nevertheless, even individuals who purchased iodized salt were not aware of many of iodine’s health benefits.

The main predictor of iodized salt acquisition was awareness of iodine’s health benefits. This finding supports the hypothesis that increasing population literacy could increase the substitution of regular salt with iodized salt. Of note, a study conducted in an Azorean island found that while lack of knowledge remained the main barrier, 47% of responders reported consuming iodized salt at home following information campaigns by the regional government [17]. This reinforces the important role of governments and health authorities in promoting literacy campaigns.

The low level of literacy was further evidenced by a perception of non-purchasers associating iodine with health hazards and attributing greater health benefits to other salt types such as salt flower or Himalayan salt, a trend also observed in other countries [21,29]. Recommendations from health professionals are cited both as reasons for purchasing and for not purchasing iodized salt. While iodine restriction may be necessary in certain rare clinical conditions, most recommendations against iodized salt in the general population are not evidence-based and may reflect misinformation or outdated guidance among healthcare providers. This highlights the need for targeted education of health professionals alongside public literacy campaigns. An ongoing European-funded project, EuThyroid2, intends to improve awareness of iodine’s role in health, although presently restricted to Cyprus, Poland, Norway, Slovenia, the United Kingdom, Bangladesh and Pakistan [30]. The findings of this project will certainly contribute to the design of public health interventions in other countries [31]. Pricing is also a concern. Despite salt being a low-cost product, regular salt is almost always cheaper than iodized salt [16], which influences consumer choice. Public health authorities advocating for iodized salt use should consider strategies to equalize the price of both types of salt.

A note is warranted regarding public health efforts to reduce dietary salt intake. Using salt as a vehicle for iodine is compatible with salt reduction goals, as iodine concentration can be readily adjusted to meet population needs considering the recommended maximum salt intake. The WHO has stated that measures to reduce salt intake and to substitute regular salt with iodized salt are compatible and has termed this approach “double-duty action” [32]. Importantly, salt iodization programs should be implemented alongside robust monitoring systems and regulatory oversight. The iodine concentration in salt should be adjusted according to the population’s salt consumption and existing dietary iodine sources. Furthermore, assessments of iodine status should account for all major iodine sources to help prevent excessive iodine intake and the potential development of iodine-induced thyroid dysfunction [33].

### Limitations

This on-site study was conducted across several retail stores in a single major Portuguese city, which may limit the generalizability of the findings. The two-month data collection period may also be influenced by seasonal variations in purchasing patterns. Most respondents were women, which is expected given their frequent role in household purchasing decisions. Furthermore, the observational design captures only in-store salt buyers and therefore may not reflect the broader population, including online purchasers, customers of other retail chains, and individuals who delegate shopping responsibilities. The cross-sectional design of the study prevents causal inference between the identified predictors and the purchase of iodized salt. The regression analysis is exploratory in nature, and the limited sample size reduces statistical power, affecting the precision of the measure.

## 5. Conclusions

Collectively, this study demonstrates the low awareness of iodized salt existence and the benefits of iodine in a large city in the North of Portugal. Additional longitudinal studies with a representative sample will inform on the generalizability of the findings at the national level. For salt to become an effective dietary source of iodine, public health authorities should consider implementing mandatory or well-structured voluntary salt iodization programs. While literacy campaigns may be beneficial under a voluntary iodization policy, they may be costly and difficult to sustain over time and should be complemented by environmental interventions [34]. In this case, price equalization, the addition of health benefits labeling on packaging, and increased availability and visibility in retail displays should be considered.

## Figures and Tables

**Table 1:** General characteristics of supermarket inquiry responders.

Characteristics	All (n = 396)	Actual Purchasers of Iodized Salt (n = 42)	Non-Purchasers of Iodized Salt (n = 354)	*p*
Age, years [mean (SD)]	51 (15)	50 (15)	51 (15)	0.066
Female gender, %	82	86	82	0.542
Education level, %				0.161
9th grade or lower	41	31	42	
Secondary or higher	59	69	58	
Pregnancy/postpartum in close circle, %	30	36	29	0.397
Responsible for purchase, %	85	93	84	0.146
Salt bought is usual choice, %	80	81	79	0.811
Type of salt purchased, %				n/a
Coarse non-iodized salt	45	0	53	
Fine non-iodized salt	18	0	21	
Sea salt, non-iodized	17	0	20	
Iodized salt (coarse and/or fine)	10	100	0	
Himalayan salt, non-iodized	7	0	8	
Salt flower, non-iodized	3	0	3	
Recognized iodized salt, %	78	90	45	<0.001
Usually consumes iodized salt at home, %	21	86	13	<0.001
Believes iodized salt is beneficial, %				<0.001
Yes	20	60	15	
No	8	7	8	
Don’t know or n/a	72	33	77	

Abbreviations: n/a, not applicable; SD, standard deviation.

**Table 2:** Predictors of actual iodized salt purchase (n = 396).

	Simple Model			Multiple Model		
Variables	OR	95% CI	*p*	OR	95% CI	*p*
Sex (females vs. males)	0.76	0.31–1.87	0.543	1.20	0.41–3.31	0.768
Age (per year increase)	1.02	0.99–1.04	0.061	1.04	1.01–1.07	0.011
Education (9th grade or lower vs. secondary or higher)	1.63	0.82–3.24	0.164	1.40	0.65–3.60	0.328
Pregnancy/postpartum in close circle (yes vs. no)	1.34	0.68–2.61	0.398	1.53	0.71–3.14	0.292
Responsible for salt purchase (yes vs. no)	2.39	0.72–8.01	0.158	2.62	0.67–10.60	0.164
Salt bought is usual choice (yes vs. no)	1.10	0.49–2.49	0.811	1.31	0.55–3.25	0.528
Attributed health benefits to iodine (yes vs. no)	8.17	4.14–16.14	<0.001	8.77	4.14–18.60	<0.001

Abbreviations: OR, odds ratio; CI, confidence intervals.

**Table 3:** Health benefits beliefs within consumers who recognized iodized salt.

Health Benefits	All (198)	Actual Purchasers (n = 38)	Non-Purchasers (n = 160)
Agreement/disagreement, Md (P25, P75)			
“Iodized salt contributes to:”			
- the psychomotor development of children	3 (3, 4)	3 (3, 4)	3 (3, 3)
- the normal functioning of the thyroid	3 (3, 4)	4 (3, 5)	3 (3, 3)
- preventing asthma	3 (3, 3)	3 (2, 3)	3 (3, 3)
- protection from sunburn	3 (3, 3)	3 (2, 3)	3 (3, 3)
- preventing goiter	3 (3, 3)	3 (3, 4)	3 (3, 3)
- protection in nuclear accidents	3 (3, 3)	3 (3, 3)	3 (3, 3)

Abbreviations: Md, median; P25, percentile 25; P75, percentile 75.

**Table 4:** Motives to purchase iodized salt.

Motives to Purchase	%	Description	Example Quote (Translated)
Associated health benefits	62	Associates the importance of consuming iodized salt with health benefits	“Because it’s good for health, especially for the thyroid”; “It’s healthier”.
Organoleptic characteristics	9	The purchase is influenced by sensory aspects perceived (taste or texture)	“To give more flavor to the food”; “Not because it’s iodized, but because it’s finer than non-iodized salt”.
Use of iodized salt in home country	7	All salt in their country of origin is iodized	“Because in Brazil it is mandatory by law for salt to be iodized, so I buy it”.
Price	6	Mentions the influence of price on the purchase of iodized salt	“Whichever is cheaper”; “Because the price/quality ratio is the most appealing”.
Variation	5	Varies the type of salt to purchase without other motives	“I buy it from time to time”; “I like to vary”.
Recommendation by health professionals	5	Follows a medical or other health professional recommendation to purchase iodized salt	“My doctor recommended using iodized salt because I have thyroid nodules”; “The nutritionist advised using iodized salt”.
Others	6	Does not identify a specific reason or points to a unique reason	“It’s my wife who says to buy it”; “I don’t know, I don’t have a specific reason”; “My parents used to purchase”.

**Table 5:** Deterrents of iodized salt purchase.

Deterrents to Purchase	%	Description	Example Quote (Translated)
Lack of knowledge	25	Refers that do not have enough information on this type of salt, do not know it, nor its characteristics	“Lack of knowledge about the salt”; “I don’t know it well; I’ve never noticed it or seen it for sale”.
Habit	22	The ingrained habit of using other types of salt is the reason not to purchase iodized salt	“Because I’m used to the coarse salt I’ve always used”; “Because she is used to buying fine salt”.
Health benefits of other types of salt	12	States that other types of salt have health benefits or are healthier in general	“I got used to salt flower many years ago, and that’s what I use”; “It’s a habit; I’ve always used others”.
Price	11	Mentions price as the reason for not buying iodized salt	“I always go for the cheapest”; “I used it during my pregnancy, as recommended by my family doctor, but now the price hasn’t gone down, so I buy the cheapest one”.
Adverse health impact	5	Attributes health hazards to iodine consumption	“I have thyroid problems, and I can’t consume a lot of iodine”; “It’s not as healthy because of the iodine”; “Iodine makes the body retain a lot of fluids; it’s bad for one’s health”.
Lack of availability	4	Refers to the inability to find it in supermarkets	“I don’t usually see iodized salt for sale in stores”; “I never noticed it was available”.
Belief that all salt is iodized	4	Believes that all salt is iodized and purchased non-iodized salt without realizing it	“I think all salt is iodized”; “I don’t know which one it is. In Brazil, all salt is iodized”.
Organoleptic characteristics	2	Indicates that sensory aspects perceived (taste or texture) influence the purchase of other types of salt	“I use sea salt. Iodized salt seems to be moister; I prefer something lighter that doesn’t retain moisture”; “I have no reason; I’ve never tried it. I’m satisfied with this sea salt; it doesn’t make the food as salty”.
Health professional recommendation	4	Follows a medical or other health-professional recommendation to purchase other types of salt or to avoid iodized salt	“I don’t know the taste. I used to buy coarse salt, but the doctor recommended Himalayan salt because it’s better for your health”.
Others	11	Does not identify a specific reason or points to a unique reason	“I don’t have a motive”; “Because as far as I know, I don’t need iodine, I already get iodine from seafood”.

## Data Availability

Data are available upon reasonable request to the authors.

## Abbreviations

The following abbreviations are used in this manuscript:

WHO	World Health Organization
TSH	Thyroid-stimulating hormone
OR	Odds ratio
CI	Confidence intervals

## Appendix A

**Table A1:** In-store questionnaire, translated to English.

Knowledge, Motives to Purchase and Beliefs About Iodized Salt					
-Can you please tell me which salt(s) you purchased?					
-Are you the person responsible for purchasing salt in your household? 1—Yes 2—Not always (Who else usually purchases salt for your household?) 3—No (Who usually purchases salt for your household?)					
-Is this the salt you usually purchase? 1—Yes 2—No (Which salt(s) do you usually purchase?) 9—I don’t know					
-Please tell me three types of salt you can recall:					
1— 2— 3—					
-Which of the following types of salt listed do you know or have heard of? Note: random order 1—Sea salt 2—Himalayan salt 3—Iodized salt 4—Salt Flower 5—Coarse salt 6—Fine salt 7—Other (Which?)					
-Do you usually consume iodized salt at home? 1—Yes (Can you please tell me the main reason for purchasing iodized salt?) 2—No (Can you please tell me the main reason for not purchasing iodized salt?) 9—I don’t know					
-In your opinion, is there any benefit or situation where consuming iodized salt is beneficial? 1—Yes (Can you please tell me what the benefits are or in what situation it might be beneficial to consume iodized salt?) 2—No 9—I don’t know/Not applicable					
-I would now like you to indicate your level of agreement with the statements I will read. Iodized salt contributes to… [Read statement] Would you say you strongly disagree (SD), disagree (D), neither agree nor disagree (NAND), agree (A), or strongly agree (SA)? Statement	SD	D	NAND	A	SA
-… the psychomotor development of children.	1	2	3	4	5
-… the normal functioning of the thyroid.	1	2	3	4	5
-… preventing asthma.	1	2	3	4	5
-… protection from sunburn.	1	2	3	4	5
-… preventing goiter.	1	2	3	4	5
-… protection in nuclear accidents.	1	2	3	4	5
To conclude, I would like you to indicate… -Gender 1—Male 2—Female 3—Other 9—No response					
-What year were you born?					
-What is your highest completed level of education? 0—Cannot read/write 1—Can read and write/Did not complete primary education 2—1st Cycle—Completed Primary Education 3—2nd Cycle—6th grade/Former 2nd year (completed) 4—3rd Cycle—9th grade/Former 5th year (completed) 5—Secondary—11th/12th grade/Former 7th year (completed) 6—Bachelor’s or Licentiate degree 7—Master’s degree 8—Doctorate					
-In your group of friends and family, is there any ongoing pregnancy or recent birth? 1—Yes 2—No 9—Don’t know					
